# Caffeine-Induced Ca^2+^ Oscillations in Type I Horizontal Cells of the Carp Retina and the Contribution of the Store-Operated Ca^2+^ Entry Pathway

**DOI:** 10.1371/journal.pone.0100095

**Published:** 2014-06-11

**Authors:** Ting Lv, Hai-Qing Gong, Pei-Ji Liang

**Affiliations:** School of Biomedical Engineering, Shanghai Jiao Tong University, Shanghai, China; Dalhousie University, Canada

## Abstract

The mechanisms of release, depletion, and refilling of endoplasmic reticulum (ER) Ca^2+^ were investigated in type I horizontal cells of the carp retina using a fluo-3-based Ca^2+^ imaging technique. Exogenous application of caffeine, a ryanodine receptor agonist, induced oscillatory intracellular free Ca^2+^ concentration ([Ca^2+^]_i_) responses in a duration- and concentration-dependent manner. In Ca^2+^-free Ringer’s solution, [Ca^2+^]_i_ transients could also be induced by a brief caffeine application, whereas subsequent caffeine application induced no [Ca^2+^]_i_ increase, which implied that extracellular Ca^2+^ was required for ER refilling, confirming the necessity of a Ca^2+^ influx pathway for ER refilling. Depletion of ER Ca^2+^ by thapsigargin triggered a Ca^2+^ influx which could be blocked by the store-operated channel inhibitor 2-APB, which proved the existence of the store-operated Ca^2+^ entry pathway. Taken together, these results suggested that after being depleted by caffeine, the ER was replenished by Ca^2+^ influx via store-operated channels. These results reveal the fine modulation of ER Ca^2+^ signaling, and the activation of the store-operated Ca^2+^ entry pathway guarantees the replenishment of the ER so that the cell can be ready for response to the subsequent stimulus.

## Introduction

Ca^2+^ is a ubiquitous intracellular messenger that regulates numerous cellular processes including muscle contraction, transmitter release, apoptosis, and so on [1], [2]. In neurons, the basal level of intracellular free Ca^2+^ concentration ([Ca^2+^]_i_) is maintained very low [3]. When activated by proper stimulation, the opening of the plasma membrane Ca^2+^ channels or the activation of Ca^2+^ release channels on the intracellular Ca^2+^ stores (largely the endoplasmic reticulum, ER) leads to the elevation of [Ca^2+^]_i_ [2].

As the primary intracellular reservoir of Ca^2+^ and a major source of [Ca^2+^]_i_ elevation, the ER is involved in a wide range of neuronal Ca^2+^-dependent processes, such as synaptic transmission and plasticity [4], [5]. The ER accumulates Ca^2+^ by active transport of Ca^2+^ from the cytoplasm into the ER by Ca^2+^-ATPase (sarco/endoplasmic reticulum Ca^2+^-ATPase, SERCA) pumps expressed on the ER membrane. At the meantime, Ca^2+^ release from the ER is mainly mediated by two families of Ca^2+^ release channels, i.e., the inositol 1,4,5-trisphosphate receptor (IP_3_R) and ryanodine receptor (RyR) families. While IP_3_Rs are gated by IP_3_, both IP_3_Rs and RyRs can be activated by Ca^2+^, and such Ca^2+^-induced Ca^2+^ release (CICR) forms a positive feedback process. RyRs can also be activated by caffeine, which sensitizes RyR’s response to Ca^2+^ [6], [7].

Type I horizontal cells (H1 HCs) are interneurons in the outer retina of carp, which receive glutamate input from cone photoreceptors. It was found in the fish retina that HCs contained caffeine-sensitive ER [8], [9] which was involved in the modulation of synaptic strength [10], GABA transporter currents [11], [12], as well as voltage-gated Ca^2+^ channel currents [13]. Despite its functional significance, knowledge about the ER Ca^2+^ dynamics of H1 HCs is still limited.

The present study aims to investigate possible mechanisms of ER Ca^2+^ dynamics of carp H1 HCs, particularly the details of the following aspects: (1) the temporal characteristics of [Ca^2+^]_i_ signals initiated by ER Ca^2+^ release, (2) the inter-relationship between the two major Ca^2+^ sources, i.e., the extracellular Ca^2+^ and the ER, and relevant channels underlying their interaction.

To explore the above issues, Ca^2+^ signals elicited by exogenously applied caffeine were recorded from freshly dissociated H1 HCs using a fluo-3-based Ca^2+^ imaging technique. The basic findings are: (1) caffeine induced oscillatory [Ca^2+^]_i_ responses in a duration- and concentration-dependent manner, (2) removal of extracellular Ca^2+^ abolished the repeatability of caffeine-induced [Ca^2+^]_i_ responses, (3) inhibition of L-type voltage-gated Ca^2+^ channels (L-VGCCs) reduced caffeine-induced [Ca^2+^]_i_ oscillations, (4) inhibition of store-operated channels (SOCs) abolished caffeine-induced [Ca^2+^]_i_ oscillations. These results reveal the fine modulation of ER Ca^2+^ signaling, and the activation of the store-operated Ca^2+^ entry (SOCE) pathway guarantees the replenishment of the ER so that the cell can be ready for response to the subsequent stimulus.

## Materials and Methods

### Ethics Statement

The animal experiments were approved by the Ethic Committee, School of Biomedical Engineering, Shanghai Jiao Tong University. All procedures strictly conformed to the humane treatment and use of animals as prescribed by the Association for Research in Vision and Ophthalmology.

### Cell Isolation

H1 HCs were enzymatically dissociated from retinas of adult carp (*Carassius auratus*, 15–20 cm body length). After 30 min dark-adaption, the eye was enucleated and hemisected. The retina was then isolated and cut into 8–12 pieces and incubated for 30 min at room temperature of 25°C in 5 ml Hank’s solution (see below) containing 25 U/ml papain (E. Merck, Germany) and 1 mg/ml L-cysteine. The retinal pieces were then kept in normal Hank’s solution at 4°C until being used (within 4 h). To obtain dissociated H1 HCs, retinal pieces were gently triturated with fire-polished glass pipettes in normal Ringer’s solution. The cell suspension was moved to a recording chamber for Ca^2+^ imaging recording.

### Solutions

Hank’s solution contained (in mM): 137.0 NaCl, 3.0 KCl, 1.0 MgSO_4_, 1.0 NaH_2_PO_4,_ 0.5 NaHCO_3_, 2.0 CaCl_2_, 2.0 Na-pyruvate, 20.0 HEPES and 16.0 Glucose. Ringer’s solution contained (in mM): 145.0 NaCl, 5.0 KCl, 1.0 MgSO_4,_ 2.0 CaCl_2_, 10.0 HEPES and 16.0 Glucose. Ca^2+^-free Ringer’s solution was prepared based on normal Ringer’s solution with CaCl_2_ omitted and 1 mM EGTA added. Caffeine was directly dissolved in Ringer’s solution according to the concentration required. Ryanodine (Tocris Bioscience, UK), nifedipine, thapsigargin (TG) and 2-aminoethoxydiphenyl borate (2-APB) were prepared in dimethyl sulfoxide (DMSO) and diluted to their final concentration in Ringer’s solution (DMSO <0.5%). The pH value was adjusted to 7.4 with NaOH for Ringer’s and Hank’s solutions as well as other solutions. All drugs, unless otherwise specified, were purchased from Sigma Aldrich (St. Louis, MO).

### Ca^2+^ Imaging

[Ca^2+^]_i_ changes were measured using a fluo-3 imaging system. Fluo-3/AM was dissolved in DMSO (1 mM stock solution) and added to the cell suspension at a final concentration of 5 µM (DMSO = 0.5%). Cell suspension was transferred into a perfusion chamber (RC-26, Warner Instruments, USA) with a cover glass bottom (12–548B, Thermo Fisher Scientific Inc., USA) and incubated at room temperature (25°C) for 15 min to allow for cell adherence and fluo-3 loading. The cells were then continuously perfused with Ringer’s solution at a flow rate of 1 ml/min for 10 min prior to recording. The volume of the perfusion chamber was about 500 µl. All drugs were applied by perfusion. At the perfusion rate employed, 1 min was needed for the drug to reach the perfusion chamber. Fluorescence measurements were performed on an upright fluorescence microscope (BX51WI, Olympus, Japan). H1 HCs were identified by their characteristic morphology as having a round soma with 4–8 extended, subtle dendrites [14]. Fluo-3 in its Ca^2+^-bound form was excited at 488 nm, and fluorescence emission at 525 nm was acquired every 2 s by a digital CCD camera (CoolSNAP ES^2^, Photometrics, USA). [Ca^2+^]_i_ signals were presented by the fluorescence intensity F normalized to the baseline fluorescence value F_0_ (F/F_0_). For caffeine-induced [Ca^2+^]_i_ responses, [Ca^2+^]_i_ signals were normalized against the amplitude of the first [Ca^2+^]_i_ transient. A transient peak was considered a caffeine-induced [Ca^2+^]_i_ transient if it had an amplitude larger than 3×SD of data recorded in the 1 min duration prior to caffeine application.

### Statistical Analyses

Statistics were performed using SPSS software (version 17.0, SPSS Inc., Chicago, IL, USA). Values are presented as mean ± SEM. To determine statistical significance, independent-samples *t* test and one-way analysis of variance (ANOVA) were used for comparing the results between two groups and that among multiple groups respectively. If a significant *p* value was obtained for ANOVA, *post hoc* analyses were performed using Student-Newman-Keuls (SNK) test, *p*<0.05 indicates significant differences.

## Results

### Caffeine Induced [Ca^2+^]_i_ Responses in a Duration- and Concentration-dependent Manner

Caffeine has long been used as a RyR agonist for studying RyR-mediated Ca^2+^ release from intracellular Ca^2+^ stores [15], [16]. In our present study, to investigate the temporal characteristics of [Ca^2+^]_i_ signal initiated by ER Ca^2+^ release, caffeine was applied at six different concentrations (1, 3, 6, 10, 20, and 40 mM) in combination with four different durations (30, 60, 90, and 240 s). For each of these 6×4 = 24 combinations, one group of independent experiments was conducted (using 5–9 H1 HCs for each experimental condition).

To study the effect of duration of caffeine application, the four groups of experiments with varying durations (i.e., 30, 60, 90, and 240 s) of caffeine application at the same caffeine concentration were compared.

In our experiments, the application of 1 mM caffeine with durations of 30, 60, 90, and 240 s induced no discernible [Ca^2+^]_i_ changes in all four groups of H1 HCs tested.

**Figure 1:**
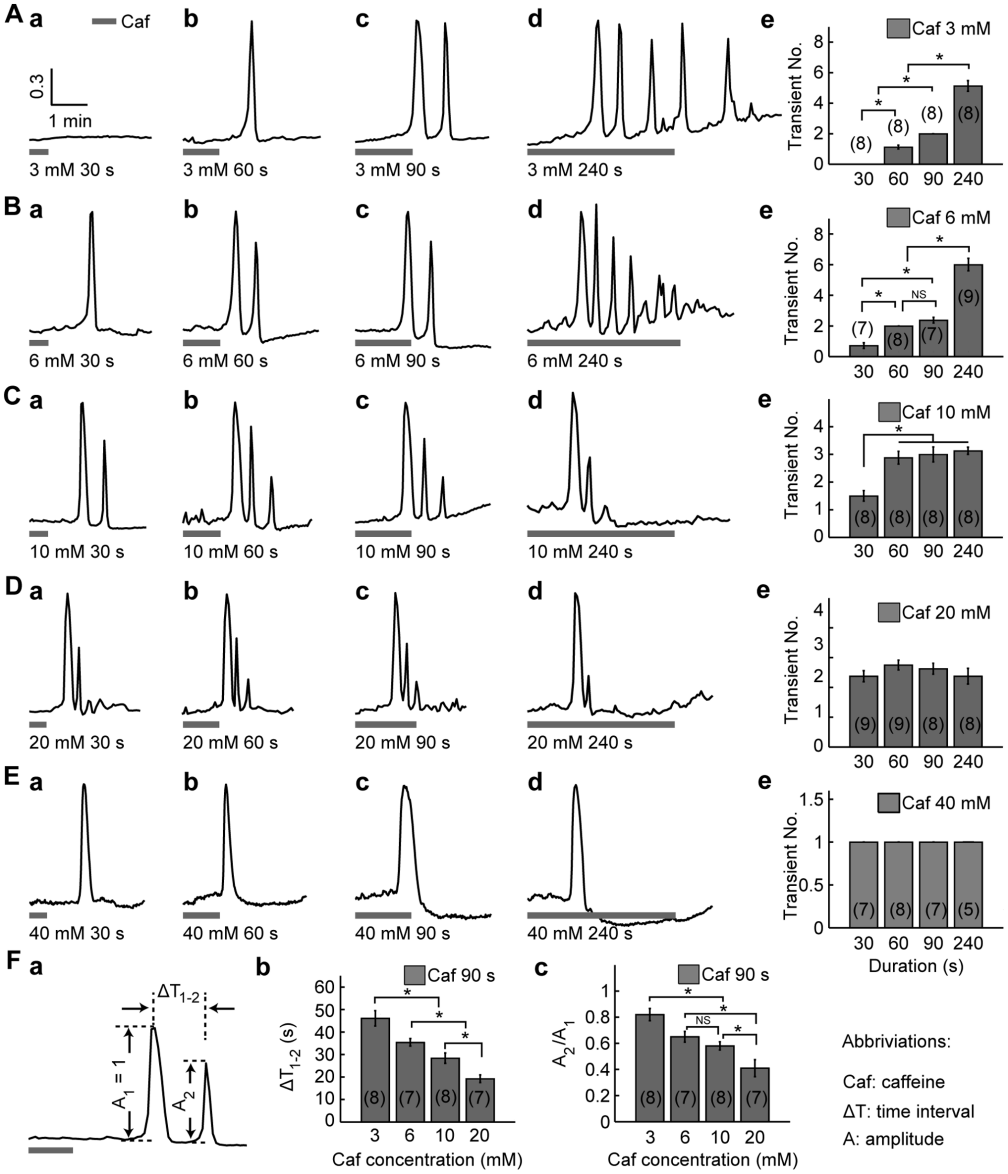
Caffeine induced [Ca^2+^]_i_ increase in a duration- and concentration-dependent manner. A–E, [Ca^2+^]_i_ transients induced by the application of 3, 6, 10, 20, and 40 mM caffeine, respectively. Columns (a)-(d), [Ca^2+^]_i_ transients induced by the application of caffeine for 30, 60, 90, and 240 s, respectively. Column (e), The average number of [Ca^2+^]_i_ transients (transient NO.) induced by various durations of caffeine application under each concentration. F, Dependence of caffeine-induced [Ca^2+^]_i_ responses (with identical application time of 90 s) on caffeine concentration. (a), Definition of amplitude and time interval between the first and the second Ca^2+^ peaks (ΔT_1–2_). The amplitude of the second [Ca^2+^]_i_ transient (A_2_) is normalized against that of the first one (A_1_). (b), ΔT_1–2_ decreased with caffeine concentration. (c), A_2_/A_1_ decreased with caffeine concentration. Horizontal bars bellow the traces indicate the periods of caffeine applications. With our perfusion rate being 1 ml/min, 1 min was needed for the drug to reach the perfusion chamber. In this and subsequent figures, each trace is the representative of a group of independent experiments, and data are presented as mean ± SEM (with sample size in parentheses). * denotes statistical significance of *p*<0.05 by one-way ANOVA followed by *post hoc* SNK test, NS: not significant.

The application of 3 mM caffeine induced [Ca^2+^]_i_ transient(s) in a duration-dependent way (Fig. 1A (a–d)). When comparing results from the four duration groups (30, 60, 90, and 240 s), the number of [Ca^2+^]_i_ transients elicited by 3 mM caffeine showed an increasing tendency with the duration of caffeine application, which was increased monotonically from 0 to 5.13±0.35 (*p*<0.05, ANOVA, *post hoc* SNK test) when the duration was increased from 30 to 240 s (Fig. 1A (e)).

The application of 6 mM caffeine tended to induce more active [Ca^2+^]_i_ transients (Fig. 1B (a–d)). The number of [Ca^2+^]_i_ transients elicited by 6 mM caffeine was increased from 0.71±0.18 to 6.00±0.41 (*p*<0.05, ANOVA, *post hoc* SNK test), when the duration of caffeine application was increased from 30 to 240 s (Fig. 1B (e)).

When 10 mM caffeine was applied, duration-dependent [Ca^2+^]_i_ transients increment was still observed (Fig. 1C (a–d)). But the increment was very much limited. The number of [Ca^2+^]_i_ transients induced by 10 mM caffeine was increased from 1.50±0.19 to 2.88±0.23 (*p*<0.05, ANOVA, *post hoc* SNK test; Fig. 1C (e)) when the duration of caffeine application was increased from 30 to 60 s, and was stabilized around 3 when the duration of caffeine was further increased to 90 and 240 s (3.00±0.27 and 3.13±0.13, respectively), with no statistical significance among the 60, 90, and 240 s groups (*p*>0.05, *post hoc* SNK test; Fig. 1C (e)).

When 20 mM caffeine was applied at various durations (30, 60, 90, and 240 s), the pattern of [Ca^2+^]_i_ response was almost uniform among the four groups (Fig. 1D (a–d)), the average number of [Ca^2+^]_i_ transients for the four groups were 2.38±0.18, 2.75±0.16, 2.63±0.18, and 2.38±0.26, respectively, with no significant differences among groups (*p*>0.05, ANOVA, *post hoc* SNK test; Fig. 1D (e)).

During 40 mM caffeine application, only one [Ca^2+^]_i_ transient could be induced no matter how long the caffeine application duration was (Fig. 1E).

The above results show that, Ca^2+^ oscillations could be induced by intermediate concentrations of caffeine (3 to 20 mM), with the oscillatory behavior being concentration-dependent. To examine the effect of caffeine concentration on Ca^2+^ oscillations, experiments conducted at varying caffeine concentrations with identical duration (90 s) of caffeine application were compared (Fig.1 column c). When the concentration of caffeine was increased from 3 to 20 mM (with identical application time of 90 s), the time interval between the first and the second Ca^2+^ transients (ΔT_1–2_; Fig. 1F (a)) decreased from 46.08±3.41 to 19.24±1.68 s (*p*<0.05, ANOVA, *post hoc* SNK test; Fig. 1F (b)).

The amplitude decrement during Ca^2+^ oscillations was also concentration dependent. During the application of 3 mM caffeine, the amplitudes of the oscillatory [Ca^2+^]_i_ transients were changing within a limited range (Fig. 1A). However, when caffeine was applied with higher concentrations, a decreasing tendency in amplitudes was observed in the oscillatory transients (Fig. 1B–D). To quantify such amplitude decrement, we normalized the amplitude of the second transient against that of the first one (A_2_/A_1_; Fig. 1F(a)). When the concentration of caffeine was increased from 3 to 20 mM (with application time of 90 s), the normalized amplitude of the second transient (A_2_/A_1_) was decreased from 0.82±0.05 to 0.41±0.07 (*p*<0.05, ANOVA, *post hoc* SNK test; Fig. 1F(c)).

Caffeine-induced [Ca^2+^]_i_ transient is composed of a rising phase and a decaying phase. In carp HCs, while the [Ca^2+^]_i_ increase is caused primarily by Ca^2+^ release from the ER via RyRs and Ca^2+^ influx from the extracellular environment, the decaying phase of the transient depends on the activity of SERCA pumps [3], [8], [9], Na^+^/Ca^2+^ exchangers [17] and plasma membrane Ca^2+^-ATPase (PMCA) pumps [17]. The balance between these Ca^2+^-increasing and removal processes determines the pattern of [Ca^2+^]_i_ oscillations, which is reflected in the time interval between two adjacent transients, and the amplitude of each transient. To investigate the underlying mechanisms of caffeine-induced [Ca^2+^]_i_ oscillations, we looked into the involvement of RyR activation in the initiation of the [Ca^2+^]_i_ oscillations.

### Ryanodine Application Inhibited the Caffeine-induced [Ca^2+^]_i_ Oscillations

**Figure 2:**
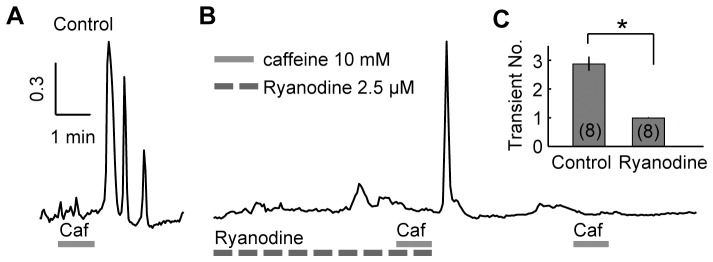
Ryanodine effect on the caffeine-induced [Ca^2+^]_i_ oscillations. A–B, Caffeine (10 mM, 60 s) induced [Ca^2+^]_i_ responses in: (A) normal Ringer’s solution (control), and (B) in the presence of ryanodine (2.5 µM). C, The average number of Ca^2+^ transients induced by caffeine (10 mM, 60 s) in the control group and the ryanodine group. * denotes statistical significance of *p*<0.05 with independent-samples *t* test.

Caffeine-induced [Ca^2+^]_i_ oscillations can be initiated by Ca^2+^ release from the ER via RyRs. To confirm the contribution of RyRs during caffeine-induced [Ca^2+^]_i_ oscillations, high concentration ryanodine (2.5 µM) was used to inhibit the Ca^2+^ release via RyRs [18], [19]. The group of experiments conducted with caffeine (10 mM, 60 s) in normal Ringer’s solution was taken as control. In normal Ringer’s solution, caffeine (10 mM, 60 s) induced three [Ca^2+^]_i_ transients in an example HC (Fig. 2A). However, in the presence of ryanodine, the application of caffeine (10 mM, 60 s) induced a single Ca^2+^ transient (Fig. 2B), which is in accordance with the notion that inhibition of RyRs by ryanodine requires the activation of RyRs [20]. Besides, due to the irreversibility of RyR inhibition by ryanodine [20], subsequent application of caffeine induced no [Ca^2+^]_i_ increase in the tested HC, even after washing out of ryanodine. Statistical results showed that, the number of [Ca^2+^]_i_ transients induced by caffeine (10 mM, 60 s) was 1.00±0.00 for the ryanodine group, which was significantly reduced as compared with 2.88±0.23 (*p*<0.05, independent-samples *t* test) of the control group (Fig. 2C), the absence of subsequent [Ca^2+^]_i_ transients after the inhibition of RyRs demonstrated that the generation of subsequent transients was also initiated by Ca^2+^ release via RyRs.

### Extracellular Ca^2+^ was Required for the Oscillatory Caffeine-induced [Ca^2+^]_i_ Responses

For each [Ca^2+^]_i_ transient, following Ca^2+^ release from the ER via RyR activation, the activation of SERCA pumps, Na^+^/Ca^2+^ exchangers, and PMCA pumps brought the elevated [Ca^2+^]_i_ back to the basal level. Due to the activity of PMCA pumps and Na^+^/Ca^2+^ exchangers in the plasma membrane, Ca^2+^ released from the ER cannot be fully recycled back into the ER by SERCA pumps. To counterbalance this loss of ER Ca^2+^, Ca^2+^ entry from the extracellular medium is required for the oscillatory caffeine-induced [Ca^2+^]_i_ responses. To investigate whether caffeine-induced [Ca^2+^]_i_ responses in H1 HCs were dependent on extracellular free Ca^2+^ concentration ([Ca^2+^]_o_), Ca^2+^-free Ringer’s solution was used to abolish the putative Ca^2+^ influx across the plasma membrane.

**Figure 3:**
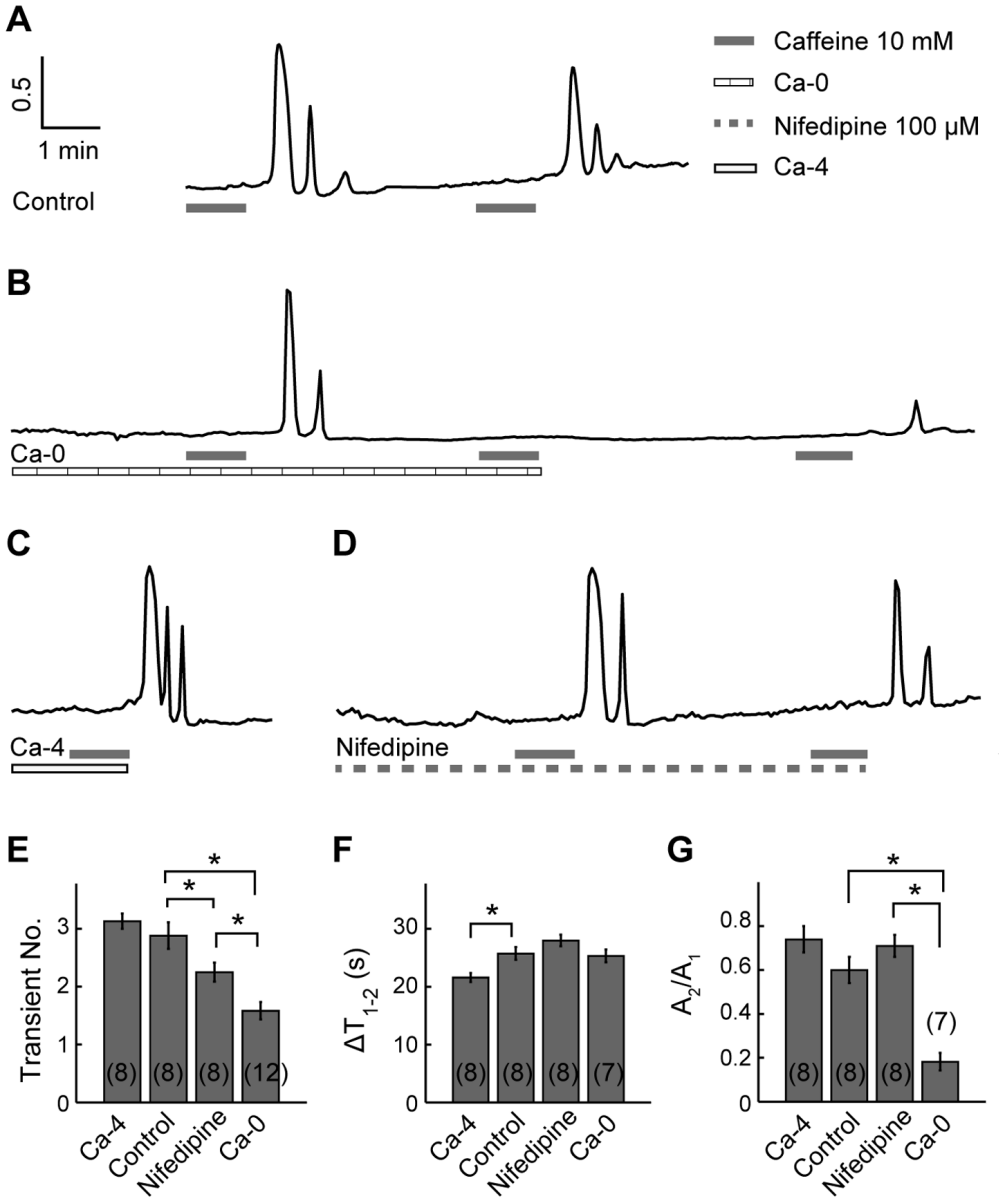
Regulation of [Ca^2+^]_o_ entry affects the caffeine-induced [Ca^2+^]_i_ oscillations. A–D, caffeine (10 mM, 60 s) induced [Ca^2+^]_i_ oscillations in: (A) normal Ringer’s solution (control) and Ringer’s solution with (B) Ca^2+^-free, (C) 4 mM [Ca^2+^]_o_, and (D) nifedipine (100 µM). E–G, The average number of [Ca^2+^]_i_ transients (E), ΔT_1–2_ (F), and A_2_/A_1_ (G) of caffeine (10 mM, 60 s) induced [Ca^2+^]_i_ oscillations for the 4 mM [Ca^2+^]_o_ group, the control group, the nifedipine group, and the Ca^2+^-free group. * denotes statistical significance of *p*<0.05 with independent-samples *t* test. Abbreviations: Ca-0: Ca^2+^-free; Ca-4∶4 mM [Ca^2+^]_o_.

The results show that, in normal Ringer’s solution (control), caffeine (10 mM, 60 s) induced three [Ca^2+^]_i_ transients in the tested HC, and such response pattern was repeatable (Fig. 3A). In Ca^2+^-free Ringer’s solution, caffeine (10 mM, 60 s) elicited two [Ca^2+^]_i_ transients, with the subsequent caffeine application inducing no measurable response (Fig. 3B), implying that the ER was depleted by the first caffeine application. After reintroduction of [Ca^2+^]_o_, caffeine (10 mM, 60 s) again induced [Ca^2+^]_i_ response, confirming the necessity of Ca^2+^ influx for ER refilling following its depletion for the subsequent Ca^2+^ response.

Of all 12 cells in the Ca^2+^-free group, caffeine (10 mM, 60 s) induced one transient in 5 cells, and two transients in the rest 7 cells. Hence, in Ca^2+^-free Ringer’s solution, the number of [Ca^2+^]_i_ transients induced by caffeine (10 mM, 60 s) was significantly reduced as compared with control (1.58±0.15 and 2.88±0.23, respectively, *p*<0.05, independent-samples *t* test; Fig. 3E). For the 7 cells of the Ca^2+^-free group in which two transients were elicited by caffeine (10 mM, 60 s), ΔT_1–2_ and A_2_/A_1_ were calculated. The ΔT_1–2_ interval was similar to that measured in control (25.33±1.10 and 25.75±1.11 for Ca^2+^-free and control group, respectively, *p*>0.05, independent-samples *t* test; Fig. 3F); however, the A_2_/A_1_ ratio was significantly smaller than that of control (0.18±0.04 and 0.60±0.06 for Ca^2+^-free and control group, respectively, *p*<0.05, independent-samples *t* test; Fig. 3G). The reduction of transients number and A_2_/A_1_ by [Ca^2+^]_o_ removal suggests that Ca^2+^ influx contributes to caffeine-induced [Ca^2+^]_i_ oscillations.

The above results show that caffeine-induced [Ca^2+^]_i_ response was significantly reduced by [Ca^2+^]_o_ removal. If abolishing Ca^2+^ entry can reduce caffeine-induced [Ca^2+^]_i_ response, up-regulation of Ca^2+^ entry should enhance the response. To test this hypothesis, [Ca^2+^]_o_ was increase from 2 mM (normal Ringer’s solution) to 4 mM to up-regulate Ca^2+^ entry from the extracellular space.

When caffeine (10 mM, 60 s) application was given in the presence of 4 mM [Ca^2+^]_o_, three [Ca^2+^]_i_ transients were evoked in an example HC (Fig. 3C). Statistical comparison between the high-[Ca^2+^]_o_ group and the control group showed that, high [Ca^2+^]_o_ resulted in a modest increase in the number of caffeine-induced [Ca^2+^]_i_ transients (2 mM [Ca^2+^]_o_: 2.88±0.23, 4 mM [Ca^2+^]_o_: 3.13±0.13, *p*>0.05, independent-samples *t* test; Fig. 3E); at the meantime, a significant decrease in ΔT_1–2_ (2 mM [Ca^2+^]_o_: 25.75±1.11, 4 mM [Ca^2+^]_o_: 21.60±0.79, *p*<0.05, independent-samples *t* test; Fig. 3F) and a modest increase in A_2_/A_1_ (2 mM [Ca^2+^]_o_: 0.60±0.06, 4 mM [Ca^2+^]_o_: 0.74±0.06, *p*>0.05, independent-samples *t* test; Fig. 3G) were also observed. Hence, up-regulation of Ca^2+^ entry indeed enhanced the oscillatory caffeine-induced [Ca^2+^]_i_ signals.

### L-VGCCs were Involved in the Caffeine-induced [Ca^2+^]_i_ Oscillations

The above results show that the caffeine-induced [Ca^2+^]_i_ response was significantly reduced when [Ca^2+^]_o_ was removed, which indicates that Ca^2+^ influx is requested for the caffeine-induced [Ca^2+^]_i_ oscillations. In carp HCs, Ca^2+^ entry from the extracellular space is known to be mediated by Ca^2+^-permeable glutamate receptors (GluRs) [8], [9], [21], [22], [23], [24] and L-VGCCs [23], [25], [26], [27]. In the present study, the activities of GluRs were precluded, because the activation of GluRs requires the binding of glutamate. On the other hand, the membrane potential and [Ca^2+^]_i_ are strongly correlated. Oscillations in [Ca^2+^]_i_ may concomitant changes of the membrane potential. Thus, L-VGCCs, which are activated by membrane depolarization, might be involved in the caffeine-induced [Ca^2+^]_i_ increase. To test the possibility of L-VGCC involvement, nifedipine, an L-VGCC antagonist, was applied [28]. Previous study in carp retinal H1 HCs suggests that 100 µM nifedipine was sufficient to abolish L-VGCC-mediated Ca^2+^ entry [23]. Hence, 100 µM nifedipine was used for complete inhibition of L-VGCCs in the present study.

In the presence of nifedipine (100 µM), caffeine (10 mM, 60 s) elicited two [Ca^2+^]_i_ transients, the subsequent caffeine application also induced two [Ca^2+^]_i_ transients (Fig. 3D). Nifedipine (100 µM) significantly reduced the number of [Ca^2+^]_i_ transients induced by caffeine (10 mM, 60 s), as compared with the effect of caffeine (10 mM, 60 s) alone (2.25±0.16 and 2.88±0.23, respectively, *p*<0.05, independent-samples *t* test; Fig. 3E). The ΔT_1–2_ interval was slightly increased (28.00±0.98 and 25.75±1.11 for nifedipine treated and control group, respectively, *p*>0.05, independent-samples *t* test; Fig. 3F), and the A_2_/A_1_ ratio was also decreased without statistical significance (0.71±0.05 and 0.60±0.06 for nifedipine treated and control group, respectively, *p*>0.05, independent-samples *t* test; Fig. 3G).

The nifedipine-induced reduction of [Ca^2+^]_i_ transient number suggested that L-VGCCs should be activated by caffeine application, and the resulting Ca^2+^ entry via L-VGCCs contributed to caffeine-induced [Ca^2+^]_i_ oscillations. However, in the presence of nifedipine, the number of [Ca^2+^]_i_ transients was significantly larger than that in Ca^2+^-free Ringer’s solution (*p*<0.05, independent-samples *t* test; Fig. 3E). Besides, nifedipine didn’t reduce the A_2_/A_1_ ratio, while the A_2_/A_1_ ratio was significantly reduced by Ca^2+^-free Ringer’s solution (Fig. 3G). More importantly, different from the result obtained with Ca^2+^-free Ringer’s solution (Fig. 3B), when caffeine was re-applied in the presence of nifedipine (Fig. 3D), [Ca^2+^]_i_ responses could be reproduced rather than abolished, which indicated that the ER could still be refilled when L-VGCCs were blocked. These differences between the effects of nifedipine and Ca^2+^-free Ringer’s solution on caffeine-induced [Ca^2+^]_i_ oscillations suggest that L-VGCCs weren’t the only Ca^2+^ entry pathway activated after caffeine application, there should be other Ca^2+^ entry pathway for ER refilling.

### SOCs were Necessary for ER Refilling

It has been reported that in some cell types, Ca^2+^ influx could be triggered by the depletion of ER Ca^2+^, a process referred to as store-operated Ca^2+^ entry (SOCE) [29], [30]. The Ca^2+^ channels mediating SOCE are called store-operated channels (SOCs).

**Figure 4:**
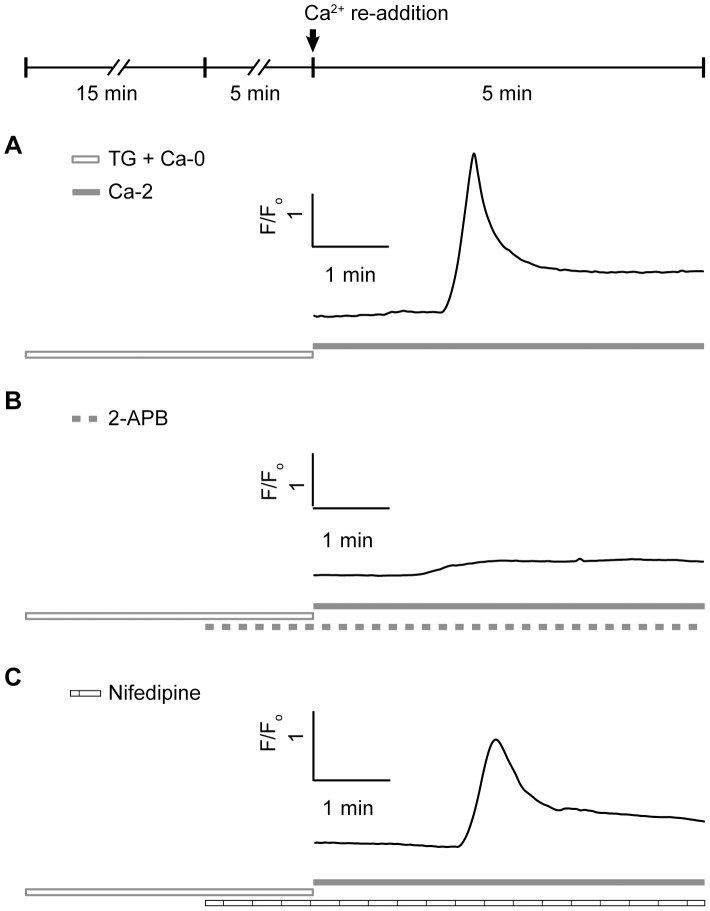
SOCs were nessicary for ER depletion-induced Ca^2+^ entry. A, After pre-application of thapsigargin (TG, 5 µM) in Ca^2+^-free Ringer’s solution for 20 min to deplete ER, reintroduction of [Ca^2+^]_o_ (2 mM) elicited a transient increase in [Ca^2+^]_i_. B, In the presence of 2-APB (a SOC inhibitor, 100 µM), the [Ca^2+^]_i_ increase induced by [Ca^2+^]_o_ re-addition was abolished. C, In the presence of nifedipine (a L-VGCC blocker, 100 µM), the [Ca^2+^]_i_ increase after [Ca^2+^]_o_ re-addition was still observed. Abbreviations: Ca-0: Ca^2+^-free; Ca-2∶2 mM [Ca^2+^]_o_. Traces shown are representative of three independent experiments, eight HCs were tested under each condition.

To examine the involvement of SOCs in ER refilling, we tested the existence of SOCs in carp H1 HCs and tried to activate SOCs by depleting the ER. To deplete the ER, HCs were incubated in Ca^2+^-free Ringer’s solution containing 5 µM thapsigargin, an irreversible SERCA inhibitor, which causes passive depletion of ER Ca^2+^ by inhibiting ER Ca^2+^ uptake via SERCA pumps [31]. If SOCs are expressed in carp H1 HCs and can be activated by ER depletion, re-addition of [Ca^2+^]_o_ should result in an increase in [Ca^2+^]_i_, given that ER Ca^2+^ uptake via SERCA pumps was inhibited. As shown in Figure 4A, after being pre-incubated in thapsigargin-containing (5 µM) Ca^2+^-free Ringer’s solution for 20 min, re-introduction of [Ca^2+^]_o_ (2 mM) caused a transient increase of [Ca^2+^]_i_ in the tested HC (Fig. 4A). Similar results were obtained from 7 others HCs, demonstrating the existence of SOCE pathways in carp H1 HCs.

To further confirm that this [Ca^2+^]_i_ increase activated by ER depletion is mediated by SOCs, 2-APB, a SOC antagonist, was applied at a concentration of 100 µM [32]. As shown in Figure 4B, in the presence of 2-APB (100 µM), [Ca^2+^]_i_ increase after [Ca^2+^]_o_ re-addition was abolished, however this [Ca^2+^]_i_ increase was not eliminated by 100 µM nifedipine (Fig. 4C). Similar results were observed from 7 other HCs for each condition. These results indicate that SOCs rather than L-VGCCs underlay this ER depletion-induced Ca^2+^ entry of HCs.

During the application of 3 mM caffeine, the number of Ca^2+^ transients was increased as the duration of caffeine was increased. At the meantime, the amplitudes of the oscillatory [Ca^2+^]_i_ transients were changing within a limited range (Fig. 1A), suggesting the occurrence of ER Ca^2+^ refilling between two adjacent [Ca^2+^]_i_ transients. To test whether SOCs underlie ER refilling after caffeine (3 mM) application, 2-APB (100 µM) was applied.

**Figure 5:**
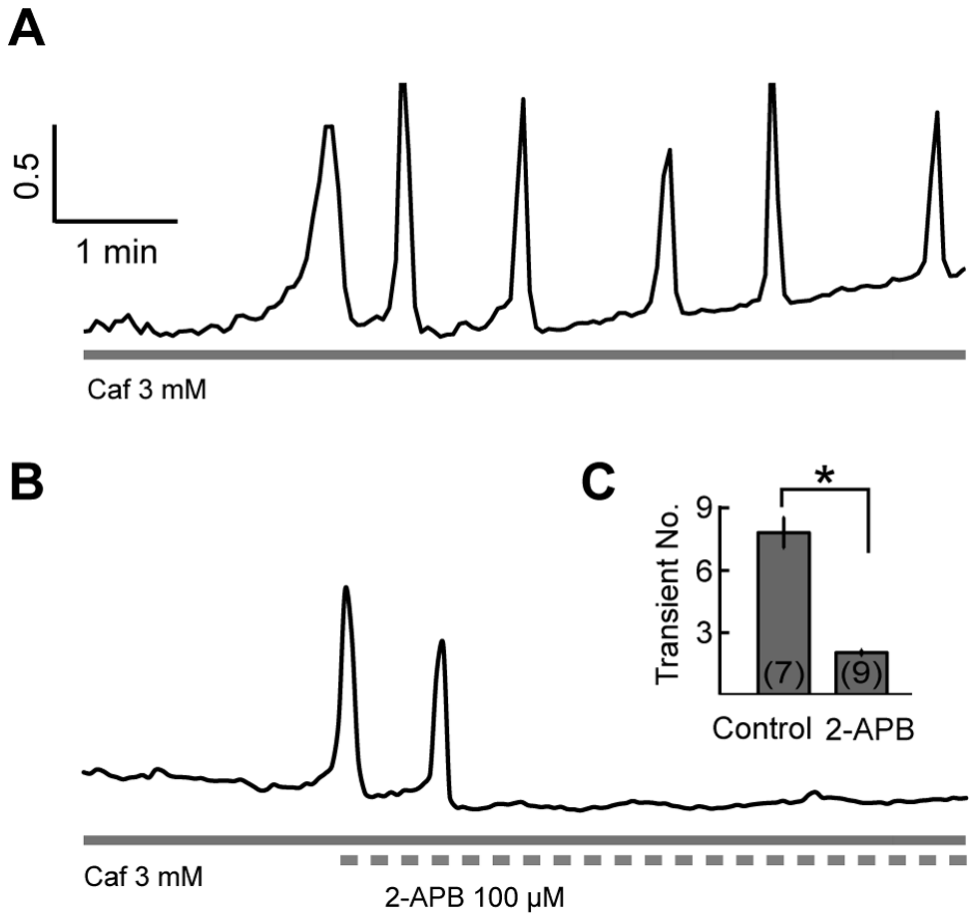
2-APB effect on the caffeine-induced [Ca^2+^]_i_ oscillations. A, [Ca^2+^]_i_ oscillations induced by prolonged application of caffeine (3 mM, 7 min). B, When 2-APB (100 µM) was co-applied, [Ca^2+^]_i_ oscillations elicited by 3 mM caffeine was abolished. C, The average number of Ca^2+^ transients induced by caffeine (3 mM, 7 min) in the control group and the 2-APB group. * denotes statistical significance of *p*<0.05 with independent-samples *t* test.

In normal Ringer’s solution, [Ca^2+^]_i_ oscillations were induced by prolonged application of 3 mM caffeine (7 min; Fig. 5A). However, such caffeine-induced [Ca^2+^]_i_ oscillations were abolished when 2-APB (100 µM) was co-applied after 2 min caffeine application (Fig. 5B). For 2-APB treated group, the number of [Ca^2+^]_i_ transients induced by caffeine (3 mM, 7 min) was 2.22±0.15, which was significantly reduced as compared with 8.57±0.81 in normal Ringer’s solution (control) (*p*<0.05, independent-samples *t* test; Fig. 5C).

Taken together, these results show that SOCs are expressed in carp H1 HCs, and can be activated by thapsigargin-induced ER depletion; when depleted by caffeine, the ER can be refilled by Ca^2+^ entry via SOCs.

## Discussion

In the present study, we investigated the mechanisms of release, depletion, and refilling of ER Ca^2+^ in carp retinal HCs. H1 HCs of carp were stimulated by caffeine under various conditions, [Ca^2+^]_i_ changes were recorded using a fluo-3-based Ca^2+^ imaging technique.

Our experimental results demonstrated that exogenous application of caffeine induced [Ca^2+^]_i_ increases in a concentration- and duration-dependent manner, with each [Ca^2+^]_i_ transient initiated by RyR activation. Extracellular Ca^2+^ was required for ER replenishment in the caffeine-induced [Ca^2+^]_i_ oscillations. Ca^2+^ influx via L-VGCCs contributed to the ER refilling, while SOCs expressed on H1 HCs were necessary for the ER refilling.

### Temporal Patterns of the Caffeine-induced [Ca^2+^]_i_ Responses

Caffeine has long been used as a RyR agonist for studying RyR-mediated Ca^2+^ release from the intracellular Ca^2+^ stores [15], [16], and caffeine-induced Ca^2+^ release has been observed in many types of neurons containing the ryanodine-sensitive ER [5]. In general, when the ER is filled with Ca^2+^, caffeine application can induce [Ca^2+^]_i_ increases even in the absence of extracellular Ca^2+^, and caffeine-induced Ca^2+^ responses can be blocked by high concentration of ryanodine. Such processes were also observed in the present study. However, the temporal characteristics of caffeine-induced Ca^2+^ responses varied with cell types. When exposed to caffeine, transient increase in [Ca^2+^]_i_ was observed in neurons such as carp retinal bipolar cells, rat spiral ganglion neurons, and rat primary sensory neurons [33], [34], [35], while sustained increase in [Ca^2+^]_i_ and [Ca^2+^]_i_ oscillations were observed in honeybee photoreceptors [36] and bullfrog sympathetic neurons [37], [38], respectively. Meanwhile, elevation in caffeine concentration (5–30 mM) increased the frequency of caffeine-induced Ca^2+^ oscillations in bullfrog sympathetic neurons. Similar to bullfrog sympathetic neurons, caffeine (3–20 mM) also induced oscillatory responses in carp H1 HCs in a concentration-dependent manner – when the concentration of caffeine was increased, the time interval between the two adjacent response peaks was decreased. However, the frequency ranges of caffeine-induced [Ca^2+^]_i_ oscillations observed in bullfrog sympathetic neurons were much lower when compared with that in carp H1 HCs. On the other hand, while the amplitudes of oscillatory caffeine-induced [Ca^2+^]_i_ increases were basically unaltered in bullfrog sympathetic neurons during the time course of caffeine application, a decreasing tendency was observed in the amplitudes of the oscillatory caffeine-induced [Ca^2+^]_i_ increases in carp H1 HCs, such differences might result from the differences in frequency ranges. At low oscillation frequencies, depleted ER might be fully refilled, so the amplitudes of the oscillatory caffeine-induced [Ca^2+^]_i_ increases were basically unaltered in bullfrog sympathetic neurons, while that measured in carp H1 HCs in our experiments had a decreasing tendency.

### The Molecular Mechanisms of SOCE

SOCE is a process by which the depletion of ER Ca^2+^ activates Ca^2+^ influx across the plasma membrane (PM) [30], the concept of which was proposed by Putney in 1986 [29]. Since then, growing evidence revealed that SOCE is a ubiquitous Ca^2+^ influx pathway that exists in a variety of cell types, including neurons [39] and non-excitable cells [40]. In recent years, it was found that stromal interaction molecule (STIM) proteins are sensors of the ER Ca^2+^ content [41], [42]. The EF-hand domain of STIM residing in the ER lumen senses the free Ca^2+^ concentration inside the lumen of the ER ([Ca^2+^]_ER_). Two types of STIM-regulated SOCs have been described: the Orai channels [43], [44], [45] and transient receptor potential canonical (TRPC) channels [46], [47]. Upon store depletion, STIM proteins and SOCs translocate and cluster at the PM-ER junctions, leading to the formation of the STIM-Orai [44], [48] and STIM-TRPC [49] complexes and SOC activation. Activated SOCs mediateCa^2+^ influx to refill depleted intracellular stores and regulate cellular processes [50], [51]. The Orai channels and TRPC channels can both be inhibited by 2-APB [32], [52], [53]. So based on our experimental results, it’s hard to tell whether it is Orai channels or TRPC channels that mediated the store-operated Ca^2+^ entry in HCs.

SERCA is one of the proteins identified as a part of the SOC macromolecular complex [54]. Experiments performed on HEK293T cells and HeLa cells demonstrated that SERCA co-localized with STIM at the PM-ER junctions following store depletion, and co-localization of SERCA with the STIM-Orai complex resulted in a tight coupling between SOCE and ER refilling, so that Ca^2+^ entry via SOCs was mainly transported into the ER [55], [56], [57]. In our current study, in the process of caffeine-induced [Ca^2+^]_i_ oscillations, ER refilling occurred during the interval between two adjacent [Ca^2+^]_i_ transients, during which [Ca^2+^]_i_ was relatively low, suggesting that the majority of Ca^2+^ entering the cytoplasm via SOCs was transported into the ER. This phenomenon reflected a coupling between the SOCs and SERCA pumps in carp H1 HCs.

In our experiments, 2-APB was used to inhibit SOCE (Fig. 4–5). Although 2-APB has been proven a reliable SOC inhibitor [32], it was also reported that 2-APB can affect hemi-gap-junction (HGJ) channels [58] and IP_3_Rs [59].

HGJ channels are expressed in fish retinal HCs, and are involved in negative feedback from HCs to cones [60], [61], [62]. These channels are gated by factors including membrane potential and [Ca^2+^]_o_ [63], [64], [65]. HGJ channels mediate inward currents at negative membrane potentials and outward currents at positive membrane potentials. Both the inward and outward currents are inhibited by high [Ca^2+^]_o_ in a concentration dependent manner [65]. In our experiments, isolated HCs had a resting membrane potential of about −70 mV, and [Ca^2+^]_o_ was remained constant at a level of 2 mM, and a small fraction of HGJ channels might be active. However, the application of 2-APB (100 µM) had no significant effect on the basal level of [Ca^2+^]_i_ (data not shown), suggesting that even if HGJ channels were active, the inward HGJ channel currents had little contribution to [Ca^2+^]_i_ responses observed in the present study. Outward HGJ channel currents with amplitudes larger than the inward currents can be evoked by membrane depolarization (beyond 10 mV) [66]. However, the caffeine-induced Ca^2+^ oscillations were abolished by inhibiting either Ca^2+^ influx or ER Ca^2+^ release. Therefore, it is unlikely that 2-APB abolished Ca^2+^ oscillations by inhibiting outward HGJ channel currents.

In regard to IP_3_Rs, no study suggests the expression of IP_3_Rs in carp retinal HCs. Besides, in experiments shown in Figure 4, given that the ER was permanently depleted by thapsigargin, IP_3_Rs which mediate Ca^2+^ release from the ER were not involved in the [Ca^2+^]_i_ increase observed after re-addition of extracellular Ca^2+^. Therefore, it is unlikely that 2-APB abolished [Ca^2+^]_i_ signals observed in our experiments by inhibiting IP_3_Rs.

Taken together, despite the fact that 2-APB affects channels other than SOCs, we can still make an inference that there exists a SOCE pathway in carp H1 HCs, which reloads the ER after its depletion.

### The Mechanism of the Caffeine-induced [Ca^2+^]_i_ Oscillations in H1 HCs

Our results showed that caffeine at low concentration (1 mM) elicited no [Ca^2+^]_i_ changes in H1 HCs, and a single [Ca^2+^]_i_ transient was evoke by high concentration (40 mM) caffeine, while [Ca^2+^]_i_ oscillations were induced by caffeine at intermediate concentrations (3 to 20 mM). The present results indicated that each [Ca^2+^]_i_ transient was initiated by RyR activation, and ER refilling, which was required for the generation of subsequent [Ca^2+^]_i_ transients, was depending on SOCE. Hence RyRs and SOCs are likely the two major components for caffeine-induced [Ca^2+^]_i_ oscillations.

Researches on caffeine effect on single RyR channel found that RyR open probability (P_o_) was increased when caffeine level was elevated [6], [67]. Low dose of caffeine (<1 mM) increased the P_o_ by increasing the opening frequency without altering the opening duration. While at high doses, caffeine elevated the P_o_ by increasing both the frequency and the duration of opening events. The relationship between RyR P_o_ and caffeine concentration could explain the dependence of the [Ca^2+^]_i_ response pattern on caffeine concentration observed in our present study. When caffeine was applied at low level (1 mM), the RyR P_o_ was very small [6]. With low RyR opening frequency and duration, caffeine was unable to trigger CICR from the ER. On the other hand, upon the application of high dose caffeine (40 mM), with RyR P_o_ significantly increased and approaching 1.0 [6], ER was constantly leaky, making it impossible to accumulate Ca^2+^ after the initial release, therefore only one Ca^2+^ transient was generated.

In addition to SOCs, Ca^2+^ influx via L-VGCCs also contributed to ER refilling. The involvement of L-VGCCs in caffeine-induced [Ca^2+^]_i_ oscillations suggests that the membrane potential of H1 HCs should be depolarized upon caffeine application. The application of caffeine triggered ER Ca^2+^ release and subsequent Ca^2+^ influx. In some cell types, the increase in [Ca^2+^]_i_ leads to the activation of several kinds of plasma membrane ion channels, including Ca^2+^-activated chloride channels, potassium channels, and non-selective channels [68], [69], [70], [71]. Since no evidence suggests that these channels are expressed in retinal horizontal cells [26], we infer that the main reason for membrane depolarization upon caffeine application was Ca^2+^ currents.

**Figure 6:**
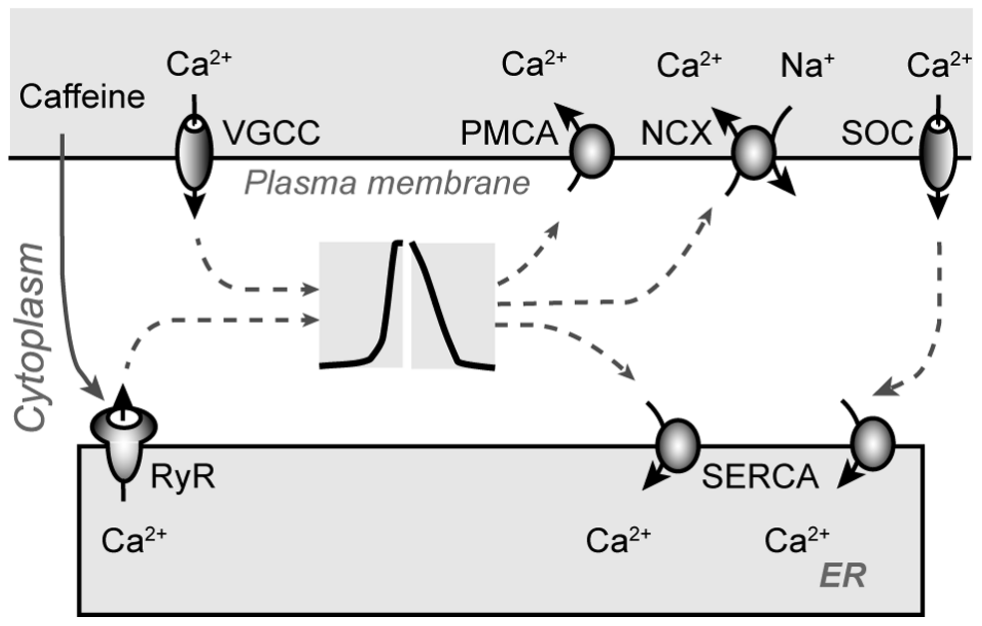
Schematic illustration of Ca^2+^ pathways relevant to the caffeine-induced [Ca^2+^]_i_ oscillations in carp retinal HCs. SOC: store-operated channel; VGCC: voltage-gated Ca^2+^ channel; PMCA: plasma membrane Ca^2+^-ATPase; NCX: Na^+^/Ca^2+^ exchanger; RyR: ryanodine receptor; SERCA: sarco/endoplasmic reticulum Ca^2+^-ATPase; ER: endoplasmic reticulum.

Taking all factors into consideration, we propose the following mechanisms underlying the caffeine-induced [Ca^2+^]_i_ oscillations (Fig. 6). When caffeine was applied at intermediate concentrations (3 to 20 mM), RyR P_o_ was at intermediate levels, which was sufficient for CICR. Upon application, caffeine increased the P_o_ of the RyR, leading to CICR from the ER. This resulted in an increase in [Ca^2+^]_i_ and ER depletion, forming the rapid increasing phase of the [Ca^2+^]_i_ transient. The elevation of [Ca^2+^]_i_ and the decreasing of [Ca^2+^]_ER_ further caused the activation of PMCA pumps, Na^+^/Ca^2+^ exchangers and SERCA pumps. PMCA pumps and Na^+^/Ca^2+^ exchangers extruded Ca^2+^ into the extracellular space, while SERCA pumps transported Ca^2+^ into the ER. The activities of these pumps and exchangers brought [Ca^2+^]_i_ back to the baseline level, forming the decay phase of the [Ca^2+^]_i_ transient, thus the first Ca^2+^ transient was generated.

Meanwhile, ER depletion activated SOCs on the PM, activated SOC mediated Ca^2+^ influx from the extracellular space. With a tight coupling between SOCE and ER refilling, Ca^2+^ influx via SOCs was mainly transported into the ER by SERCA pumps without disturbing [Ca^2+^]_i_.

Due to the activity of SERCA pumps and SOCs, the ER was refilled after the first [Ca^2+^]_i_ transient, partially or fully. At this moment, if caffeine was continuously applied, CICR could be re-activated, and a second or more [Ca^2+^]_i_ transients could be generated, thereby forming [Ca^2+^]_i_ oscillations. The higher the caffeine concentration, the larger the RyR P_o_, and the shorter it took for CICR initiation, thus ΔT_1–2_ was decreased when the caffeine concentration was increased.

The amplitudes of the subsequent [Ca^2+^]_i_ transients depend on the [Ca^2+^]_ER_ level. ΔT_1–2_ was decreased when the concentration of caffeine was increased, which means that time duration for ER refilling was decreased when the caffeine concentration was increased, therefore the A_2_/A_1_ ratio was decreased when the caffeine concentration was increased. For [Ca^2+^]_i_ oscillations induced by 3 mM caffeine, the time interval between two adjacent [Ca^2+^]_i_ transients was relatively long, which might result in the full refilling of the ER. Thus the amplitudes of Ca^2+^ transients generated during the time course of caffeine application were remained within a limited range. For [Ca^2+^]_i_ oscillations induced by higher concentrations of caffeine (6–20 mM), short interval between two adjacent [Ca^2+^]_i_ transients resulted in inadequate ER replenishment, thus the amplitudes of the oscillatory Ca^2+^ transients were gradually decreased until the ER was finally running out of Ca^2+^.

### Ca^2+^ Signaling in Carp HCs

Similar to other cell types, Ca^2+^ is an important intracellular messenger in carp HCs. [Ca^2+^]_i_ increase is involved in many HCs cellular functions, including modulation of GABA transporter currents [11], [12], modulation of VGCC currents [13], modulation of synaptic connections [10], as well as the plasticity of spinules at dendrites [21], [72], gating of HGJ channels [63], and maintenance of electrical coupling between HCs [73], etc. The universality of Ca^2+^ signaling requires the expression of many Ca^2+^-related components to create a wide range of spatially and temporally distributed signals [2].

In the vertebrate retina, glutamate is continuously released from photoreceptors in the dark, thus GluRs on HCs can be tonically activated. Activated ionotropic GluRs mediated cation influx (including Ca^2+^), leading to HC depolarization, VGCC activation, CICR from the ER and an increase in [Ca^2+^]_i_ [8], [9], [23]. A sustained high [Ca^2+^]_i_ is cytotoxic [74]. Besides, after carrying out its signaling functions, elevated [Ca^2+^]_i_ must be brought back to the basal level so that the cell can be ready for response to the subsequent stimulus. After the initial [Ca^2+^]_i_ increase, AMPA receptor desensitization [75], VGCC inhibition via store depletion [13], [76], and VGCC inhibition via AMPA receptor activation [13] might help to restrict Ca^2+^ influx, regulating [Ca^2+^]_i_ increases and CICR from the ER. Hence, oscillatory [Ca^2+^]_i_ responses, such as that observed in the present study, might be one of the strategies that HCs adopt in exposure to tonic glutamate input, so as to prevent cytotoxicity, as well as regulating [Ca^2+^]_i_ by the rate and duration of photoreceptor glutamate release.

Oscillations of the membrane potential in response to a brief light flash have been recorded from HCs in the intact retina [77], [78]. Since the membrane potential and [Ca^2+^]_i_ are strongly correlated, we infer that [Ca^2+^]_i_ oscillations can be induced under physiological conditions. But the frequency ranges of the membrane potential oscillations were between 1.5 and 3.5 Hz, much higher than the [Ca^2+^]_i_ oscillations recorded in our experiments. In our experiments, bath application of caffeine affects RyRs in isolated H1 HCs, leading to the generation of global intracellular Ca^2+^ signals. While in the intact retina, HCs receive input from synapses of rods and cones, producing localized Ca^2+^ signals. Depletion of the ER in a restricted area takes shorter time to refill, while depletion of the whole ER as observe in the current study takes longer to refill, this might account for the differences in oscillation frequencies observed in HCs in the intact retina and isolated HCs.

Generally, caffeine-induced [Ca^2+^]_i_ oscillations in carp H1 HCs is the repetition of the following two steps: ER Ca^2+^ release and subsequent ER refilling. Ca^2+^ release from the ER is mediated by RyRs, while ER refilling depends primarily on Ca^2+^ influx via SOCs. Hence the SOC is an essential component for ER refilling, and its activation is required for the maintenance of [Ca^2+^]_i_ oscillations. The ER is the primary intracellular reservoir of Ca^2+^ and a major source of [Ca^2+^]_i_ elevation, and it is involved in many cellular processes. The existence of SOCs in H1 HCs and the coupling between SOCE and ER refilling guarantee the efficient replenishment of the ER so that the cell can be ready for response to the subsequent stimulus. Thus SOCs are essential for the fulfilling of normal physiological functions of carp H1 HCs.
